# Development of prevention strategies against bath-related deaths based on epidemiological surveys of inquest records in Kagoshima Prefecture

**DOI:** 10.1038/s41598-023-29400-7

**Published:** 2023-02-08

**Authors:** Midori Katsuyama, Eri Higo, Machiko Miyamoto, Takuma Nakamae, Daiko Onitsuka, Akiko Fukumoto, Masahiko Yatsushiro, Takahito Hayashi

**Affiliations:** 1 Department of Legal Medicine, Graduate School of Medical and Dental Sciences, Kagoshima University, 8-35-1 Sakuragaoka, Kagoshima, 890-8544 Japan; 2 Kushikino Coast Guard Office, Tenth Regional Coast Guard Headquarters, Japan Coast Guard, 54-1 Urawachou, Ichikikushikino, Kagoshima 896-0036 Japan

**Keywords:** Disease prevention, Public health, Anatomy, Risk factors

## Abstract

Sudden death in the bathroom (bath-related death) occurs more frequently in Japan than in other countries. To clarify the epidemiological characteristics of bath-related deaths, we reviewed inquest records of deaths in Kagoshima Prefecture from 2006 to 2019. We identified 2689 cases of bath-related death. Of these cases, 90% were among people aged ≥ 65 years. The majority occurred in a home bathtub between 16:00 and 20:00. Most deaths (52.0%) occurred in winter (December–February), and there were extremely strong negative correlations with the environmental temperatures (maximum, minimum, and mean) on the day of death. We identified the environmental temperature during cold winter months that bath-related deaths are likely to occur in Kagoshima, although further investigation concerning the effects of other confounding factors is required. Forensic autopsies have only been performed in 29 cases and the cause of death was not diagnosed correctly in the majority of cases. Although autopsies are essential to elucidate the pathogenesis of the deaths, it is difficult to increase the rate of autopsies under the current Japanese death investigation system. Therefore, we suggest that the best way to prevent bath-related death is establishing an “Alert system” based on our results, and to have people refrain from bathing on dangerous days.

## Introduction

Sudden death in the bathroom (bath-related death) is much more common in Japan than in other countries, probably due to the unique style of bathing in Japan^1–10^. Unlike other countries, the Japanese have a bathing style in which we soak in shoulder-deep water in a deep bathtub. Especially during the cold winter months, we have a habit of soaking in hotter water (42 °C or higher) every day to warm ourselves. This custom is particularly common among the elderly. These Japanese habits are thought to be closely related to the high incidence of bath-related deaths. Actually, some reports show that bath-related deaths account for more than 10% of all sudden deaths, or approximately 14,000 cases per year in Japan^11^. The incidence rate is particularly high among people aged ≥ 65 years, and is likely to increase as the population ages, making bath-related deaths a serious social problem.

In the 23 wards of Tokyo, Osaka city, and Kobe city, where administrative autopsies are performed on bath-related deaths under the medical examiner system, the three major causes of bath-related death are acute cardiac death (i.e., ischemic heart disease), drowning, and cerebrovascular accident^12^. However, forensic autopsies are not performed on bath-related deaths in many of the remaining prefectures that do not have the medical examiner system. In such prefectures, it is left to the discretion of police whether or not to conduct a forensic autopsy, and the decision is based more on evidence of criminal death rather than determining the cause of death. To prevent bath-related death, it is essential to elucidate the pathogenesis and mechanism of the death, and to investigate the cause of death through forensic autopsy. However, it is difficult to rapidly increase the number of autopsies of bath-related deaths under the current Japanese death investigation system as described above. Therefore, an urgent alternative is needed to prevent bath-related deaths.

According to previous literature, bath-related deaths occur overwhelmingly in colder temperatures, and our previous study found similar characteristics in the warmer Kagoshima Prefecture (annual mean air temperature in Kagoshima city is 19.2 °C), located in the southwest of Japan. Although the mechanism of bath-related death has not been elucidated, several reports suggest that some bath-related deaths are induced by the difference in temperatures between the low air temperature in the bathroom and high water temperature in the bath^13,14^. Such a temperature difference may cause wild fluctuations in blood pressure, resulting in arrhythmia, ischemic heart disease, stroke, and death, or loss of consciousness followed by drowning. Therefore, we conducted a retrospective survey of inquest records of bath-related deaths in Kagoshima Prefecture that occurred during 2006–2019 to determine the epidemiological characteristics of the deaths. As mentioned above, bath-related deaths are more likely to occur in the cold winter months, and, therefore, we paid particular attention to the relationship between the occurrence of bath-related deaths and the environmental temperatures to identify the temperature at which bath-related deaths are more likely to occur. Our final goal is to develop prevention strategies to avoid bath-related deaths.

## Results

### Number of bath-related death and crude mortality rate, Age

**Figure 1:**
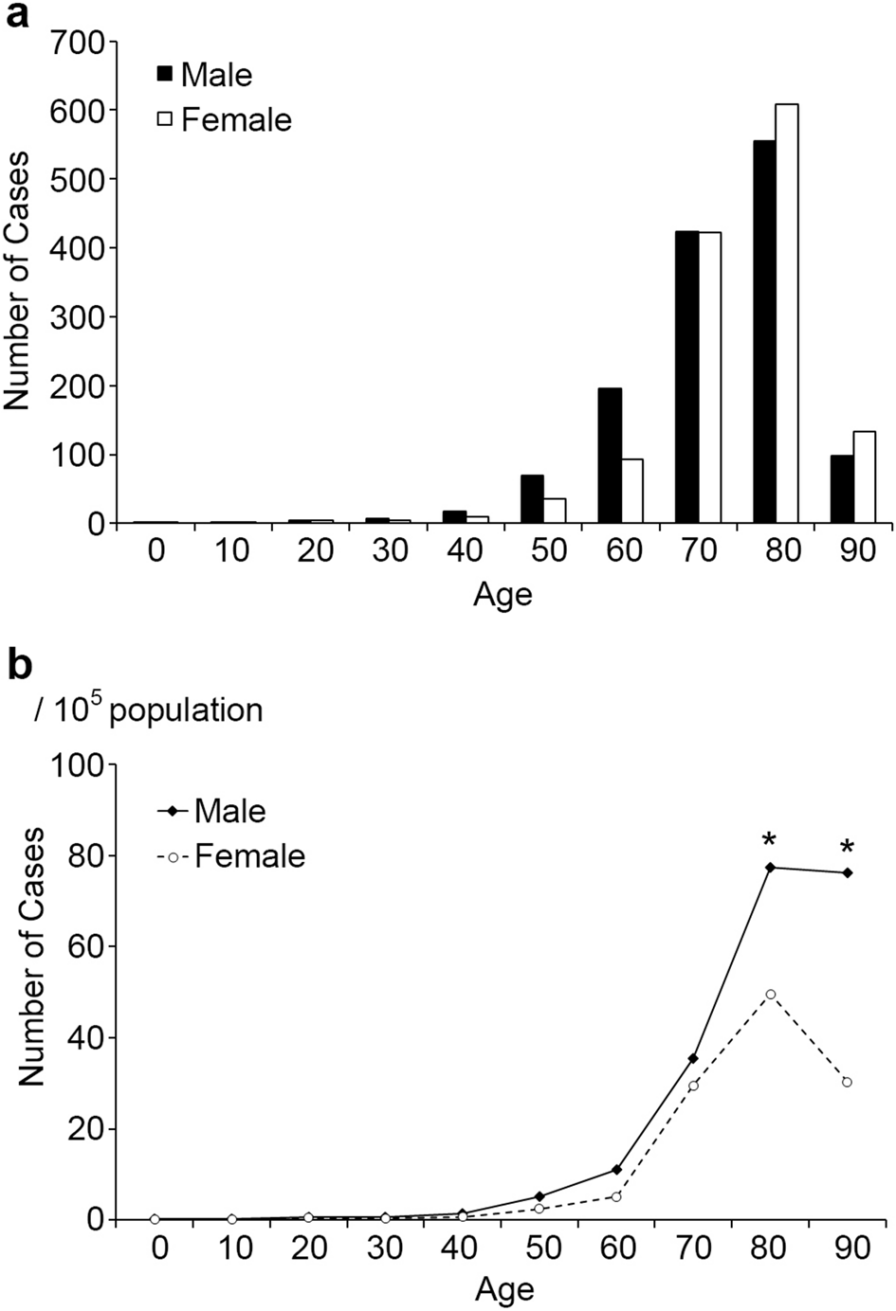
(**a**) Distribution of age and gender in bath-related death. (**b**) Crude mortality rates by age. The mortality rates were compared between men and women using chi-square (χ^2^) test under a binomial distribution of counts. *p < *0.05* men vs. women.

A total of 2689 cases of bath-related deaths (1375 men and 1314 women) were reported in Kagoshima Prefecture from 2006 to 2019 (crude yearly mortality rate, 8.9–12.7% per 100,000 individuals). No significant difference was observed in the total number of bath-related death between men and women (*p* = 0.2413), however, there were significantly higher men than women for cases > 80 years of age (*p* = 0.0472), and > 90 years of age (*p* = 0.0460). The mean age of the 2689 cases was 77.9 years, (range, 4–103 years). Of the 2689 cases of bath-related death, 2419 cases (90.0%) were ≥ 65 years old (Fig. 1a), and the mortality rate of bath-related death increased with age (Fig. 1b).

### Location of death

**Figure 2:**
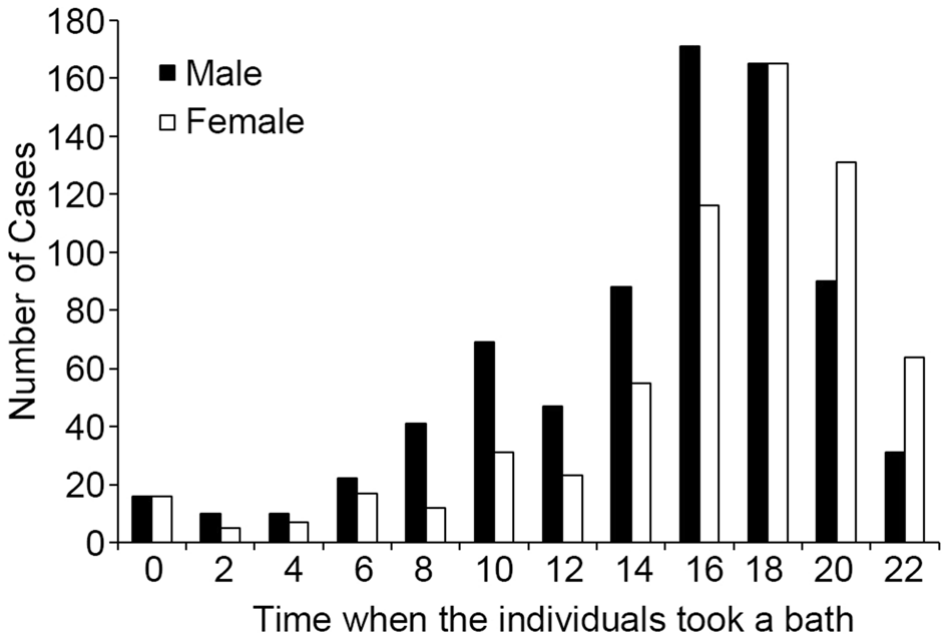
Time of the day when the victims with bath-related death took a bath.

Most of the cases of bath-related death occurred at home (n = 2292; 85.2%), followed by spas and other bathing facilities including hospitals, nursing homes, and hotels (Table 1a). In the bathroom, the bathtub was the most frequent location, occurring in 2427 cases (90.3%), followed by the space next to a bathtub in the bathroom and the dressing room (Table 1b). Most deaths were recorded between 16:00 and 20:00 (44.0%), a time when the elderly usually take a bath in Japan (Fig. 2).

**Table 1:** Location where bath-related deaths occurred.

	Male	Female	Total
(a) Types of bathroom			
Home	1067	1225	2292 (85.2%)
Spa	246	54	300 (11.2%)
Other	62	35	97 (3.6%)
	1375	1314	2689
(b) Location in the bathroom			
Bathtub	1239	1188	2427 (90.3%)
Space next to a bathtub	108	112	220 (8.2%)
Dressing room	10	11	21 (0.8%)
Other	18	3	21 (0.8%)
	1375	1314	2689

### Whether living alone or with another, Time to discovery

**Figure 3:**
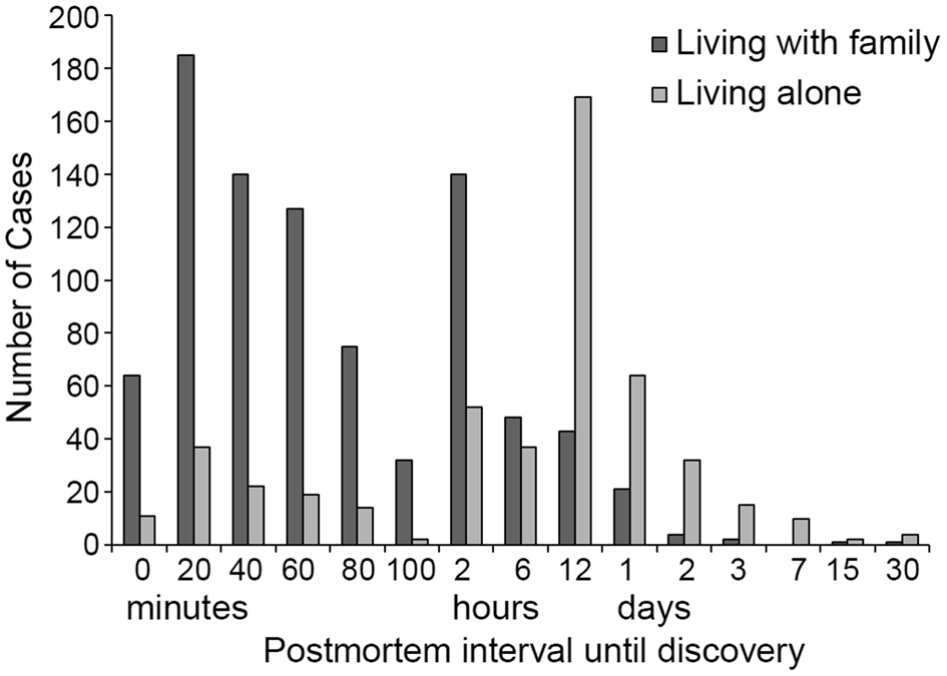
Postmortem interval until discovery. The black bars and the white bars indicate the postmortem interval until discovery in those who lived with their families and in those who lived alone, respectively. The data was extracted by bath-related death that occurred in home.

As for living style, of the 2689 cases of bath-related death, 1048 (45.9%) of the cases lived alone and 1237 cases (54.1%) lived with their family. In Kagoshima Prefecture, there were 257,593 people who lived alone and 1,401,304 people who lived with their family. Therefore, those who lived alone had a substantially higher mortality rate of bath-related death compared with those who lived with their family (406.8 vs. 88.3 individuals per million). In addition, we compared the postmortem interval until discovery for these two groups (Fig. 3). For cases who lived with their family, it took less than 2 h on average until being discovered, whereas, for cases who lived alone, it took substantially longer time to be discovered. In addition, there was no significant gender difference in the time required for discovery (men, 9.61 ± 1.93 h; women, 9.0 ± 1.29 h; *p* = 0.094).

### Presence of alcohol consumption, past history of illness

A history of alcohol drinking before bathing was reported in only 115 (4.3%) of the deaths (men, 97; women, 18). However, in 730 cases (27.1%), it was not known whether they had been drinking alcohol before bathing.

A past history of illness with incidence is presented in Table 2. Two hundred forty-two cases (9.0%) were considered to be healthy and the remaining 2447 cases had some past history of illness, including hypertension, cardiovascular disease, diabetes mellitus, and central nervous system disorder. The most common illness was hypertension (1213 cases; 45.1%), in combination with other illnesses in most cases.

**Table 2:** Past history of illness.

	Male	Female	Total
Hypertension	555	658	1213 (45.1%)
Cardiovascular diseases	329	348	677 (25.2%)
Diabetes mellitus	334	204	538 (20.0%)
CNS disorders	277	207	484 (18.0%)
Cancer	179	79	258 (9.6%)
Epilepsy	28	24	52 (1.9%)
Others	754	775	1529 (56.9%)
No history (healthy)	138	104	242 (9.0%)

*CNS* central nervous system.

### Autopsy or not, Cause of death

Surprisingly, forensic autopsies were performed in only 29 (1.08%) of the deaths. Of these 29 cases, the most common cause of death was drowning (24 cases, 82.8%), followed by ischemic heart disease (2 cases, 6.9%). In the remaining 3 cases, the cause of death was diagnosed as unknown due to advanced decomposition. Of the 24 cases diagnosed as drowning, the cause of drowning was identified in 9 cases, while the cause of drowning was unknown in the remaining 15 cases. The most common cause of drowning was cardiovascular disease (3 out of 9 cases), and other causes included metabolic disease such as liver cirrhosis, epilepsy, chronic subdural hematoma, and obstructive sleep apnea syndrome. In the other 2660 cases, no autopsy was performed and presumed the cause of death was determined only by external examination with or without postmortem CT. Postmortem CT imaging was performed in 1125 cases (56.7%), with an increasing trend year by year. The cause of death indicated on the death certificate was heart disease in 1231 cases (45.8%), drowning in 855 cases (31.8%), and central nervous system disorders such as stroke in 340 cases (12.6%). Drowning was caused by loss of consciousness due to ischemic heart disease in 15.2%, intracerebral hemorrhage and epileptic seizures in 1.8%, but most (72.7%) were unknown.

### Seasonal trend, relationship with the environmental temperatures

**Figure 4:**
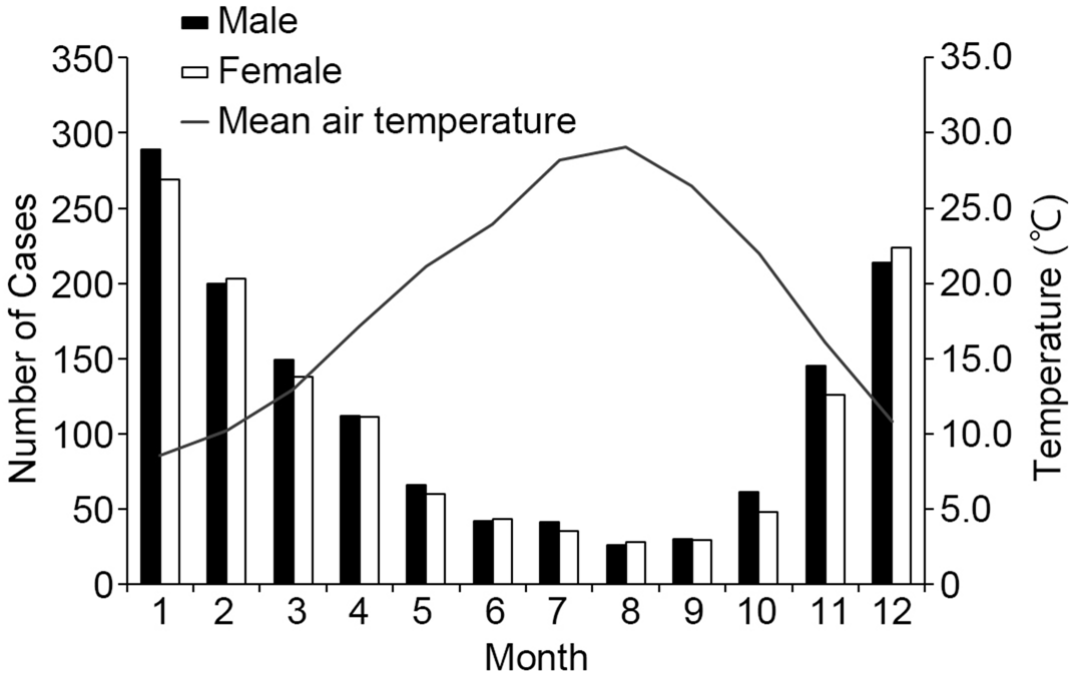
Distribution of the number of bath-related deaths by month. The seasonal trend in the occurrence in winter (December to February). Monthly distribution of the number of bath-related death and the average daily air temperature.

**Figure 5:**
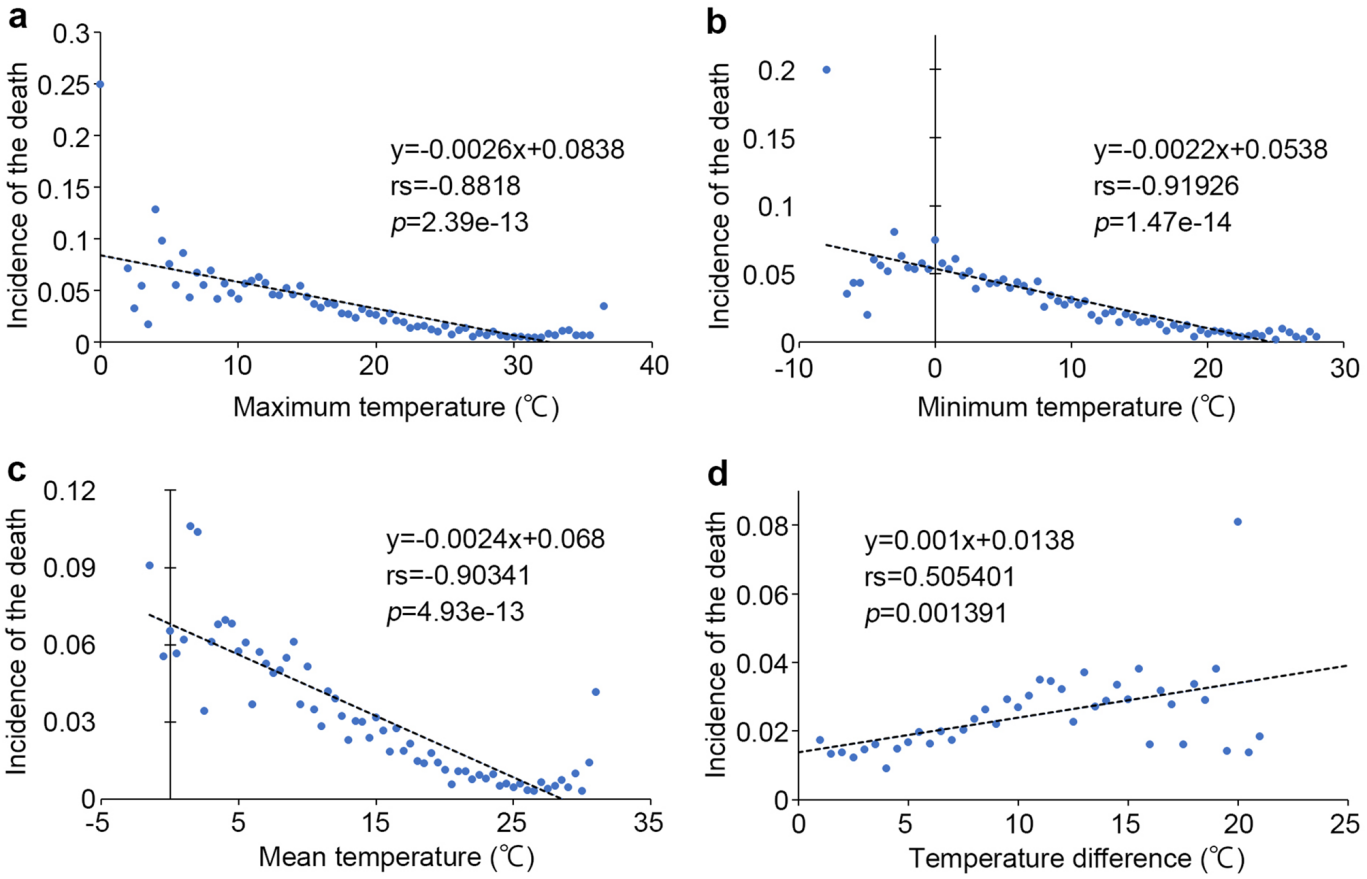
Correlation between the incidence of bath-related deaths and each environmental temperature (**a**, daily maximum temperature; **b**, daily minimum temperature; **c**, daily mean temperature; **d**, temperature difference within a day).

**Figure 6:**
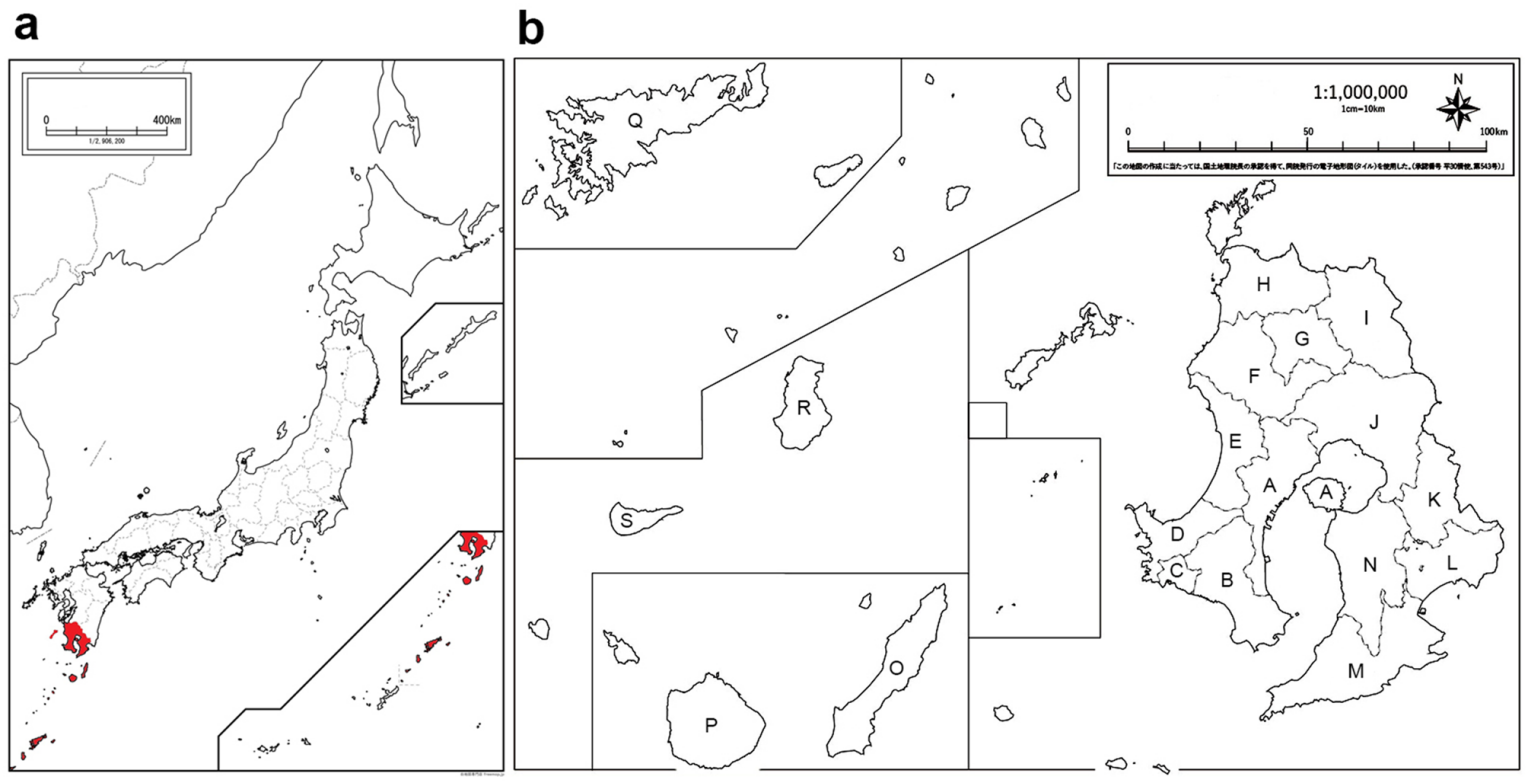
(**a**) The map of whole Japan. The area filled in red is Kagoshima Prefecture. (**b**) The map of Kagoshima prefecture. A–N, these areas are the southern tip of the main island. O–S, these areas are isolated islands. O, the location of the nearest main island (N30°43.0′ E130°58.9′). S, the location of furthermost from Kagoshima prefecture (N27°23.2′ E128°39.1′). Q, the location at the middle between O and S. The names of each subregions as follows: A, Kagoshima city; B, Ibusuki and Minamikyushu cities; C, Makurazaki city; D, Minamisatsuma city; E, Hioki and Ichikikushikino cities; F, Satsumasendai city; G, Satsuma town; H, Akune and Izumi cities; I, Isa city and Yokogawa town; J, Aira and Kirishima cities; K, Soo city; L, Shibushi city; M, Kimotsuki and Kinko towns; N, Kanoya city; O, Tanegasima (island); P, Yakushima (island); Q, Amamioshima (island); R, Tokunoshima (island); S, Okinoerabu (island).

There was a clear seasonal trend in the occurrence of bath-related death (Fig. 4). The greatest number of deaths occurred during winter months (1399 cases, 52.0% in December to February), whereas the summer months (June to August) had the least number of deaths (215 cases, 8.0%). There was no significant difference in the seasonal trend with gender. At first, the relationship between the incidence of bath-related deaths and the environmental temperatures (daily maximum temperature, daily minimum temperature, daily mean temperature, temperature difference within a day) was closely investigated. There were extremely strong negative correlations between the incidence of bath-related deaths and daily maximum temperature (correlation coefficient (rs) = − 0.8818; *p* = 2.39e−13; Fig. 5a), daily minimum temperature (rs = − 0.91926; *p* = 1.47e−14; Fig. 5b) and daily mean temperature (rs = − 0.90341; *p* = 4.93e−13; Fig. 5c). On the other hand, there was a positive correlation between the incidence of bath-related deaths and the temperature difference within a day (rs = 0.505401; *p* = 0.001391; Fig. 5d), although not as strong as for other environmental temperatures. Next, multiple regression analysis was performed to include confounding factors in the relationship between the incidence of bath-related deaths and the environmental temperatures. The confounding factors included age, male ratio, prevalence of hypertension, cardiovascular disease, diabetes mellitus, and central nervous system disorders, which were considered to contribute to the incidence of bath-related deaths in this study. The incidence of bath-related deaths was found to be influenced by male ratio and the prevalence of hypertension and diabetes mellitus at the maximum temperature, by the prevalence of hypertension and diabetes mellitus at the daily minimum temperature, and by the prevalence of central nervous system disorders at the daily mean temperature (Table 3). It was confirmed that daily maximum (t-value = − 8.4020; *p* = 7.9615e−12), minimum (t-value = − 11.1098; *p* = 1.7573e−16), and mean temperatures (t-value = − 13.3073; *p* = 3.9414e−19) all had a much greater effect on the incidence of bath-related deaths than the other factors investigated in this study. The effect of temperature differences within a day on the incidence of bath-related deaths was similar to that of the prevalence of cardiovascular disease (Table 3). These results indicate that the environmental temperature has a much greater influence on the occurrence of bath-related deaths than other confounding factors, and, therefore, a study was conducted to identify the environmental temperature at which bath-related deaths are more likely to occur. Since bath-related deaths are clearly more likely to occur in winter as mentioned above, the environmental temperatures at which bath-related deaths are more likely to occur were calculated with a focus on the winter months (December to February) as follows: daily maximum temperature of 13.5 °C or lower, daily minimum temperature of 3.5 °C or lower, daily mean temperature of 9 °C or lower, and temperature difference within a day of 8 °C or higher. Since Kagoshima Prefecture is extends a significant distance from north to south (approximately 600 km), including some remote islands, and the environmental temperature varies from region to region, we divided the Prefecture into 19 regions according to the jurisdiction of the police station that handles corpses (Fig. 6). Among the environmental temperatures, the daily maximum, minimum, and mean temperatures all showed significant negative correlations with the incidence of bath-related deaths in all regions (Supplementary Table 1). Similarly to the above, the environmental temperatures at which bath-related deaths are more likely to occur were calculated with a focus on the winter months in each region The temperature at which bath-related death were more likely to occur differed by region (maximum, 9.0–19.0 (median 13.5) °C; minimum, 0.0–13.0 (median 3.0) °C; mean, 4.5–15.5 (median 9.0) °C) (Table 4). In particular, in the remote islands located in the south of Kagoshima Prefecture (O, P, Q, R, S in Fig. 6), the temperatures at which bath-related death occurred tended to be higher than those in other regions. On the other hand, temperature difference within a day showed a significant positive correlation with the incidence of the death in only 6 regions (A, H, I, J, L, Q), but not in the remaining regions. The temperature differences within a day in the 6 regions where bath-related deaths are likely to occur were calculated to be 5.5–10.5 (median 8.8) °C or more.

**Table 3:** Multiple regression analysis of confounding factors between the incidence of bath-related deaths and each environmental temperature.

	RC	SD	SRC	PCC	t	*p*
Maximum temp						
Constant term	0.0510	0.0665	0.0510		0.7666	0.44625038
Temp	− 0.0025	0.0003	− 0.7112	− 0.7297	− 8.4020	7.9615E−12
Age	− 0.0002	0.0008	− 0.0262	− 0.0385	− 0.3030	0.76290807
Male ratio	0.0586	0.0183	0.2702	0.3778	3.2125	0.00208794
HT	0.0467	0.0208	0.2275	0.2736	2.2398	0.02870124
CVD	0.0410	0.0208	0.1723	0.2433	1.9750	0.05272799
DM	− 0.0843	0.0211	− 0.3908	− 0.4533	− 4.0045	0.00016881
CNS	0.0308	0.0214	0.1463	0.1793	1.4353	0.15622533
Minimum temp						
Constant term	0.1009	0.0460	0.1009		2.1914	0.03213183
Temp	− 0.0023	0.0002	− 0.8180	− 0.8137	− 11.1098	1.7573E-16
Age	− 0.0004	0.0006	− 0.0588	− 0.0980	− 0.7816	0.43736225
Male ratio	− 0.0197	0.0138	− 0.1210	− 0.1779	− 1.4352	0.15618143
HT	0.0272	0.0107	0.1817	0.3049	2.5409	0.01353093
CVD	− 0.0145	0.0163	− 0.0650	− 0.1114	− 0.8895	0.37712267
DM	− 0.0396	0.0186	− 0.1902	− 0.2593	− 2.1307	0.03702139
CNS	− 0.0169	0.0229	− 0.0563	− 0.0924	− 0.7364	0.46419924
Mean temp						
Constant term	0.1483	0.0470	0.1483		3.1552	0.00256094
Temp	− 0.0026	0.0002	− 0.9625	− 0.8698	− 13.3073	3.9414E-19
Age	− 0.0011	0.0006	− 0.1472	− 0.2465	− 1.9199	0.05987699
Male ratio	− 0.0060	0.0130	− 0.0343	− 0.0607	− 0.4590	0.64794913
HT	− 0.0030	0.0097	− 0.0200	− 0.0413	− 0.3121	0.75606912
CVD	0.0297	0.0167	0.1502	0.2290	1.7764	0.08099937
DM	0.0090	0.0181	0.0472	0.0660	0.4996	0.61927542
CNS	0.0317	0.0144	0.1646	0.2802	2.2035	0.03161373
Temp difference						
Constant term	− 0.0605	0.0496	− 0.0605		− 1.2195	0.23129984
Temp	0.0012	0.0003	0.6141	0.5800	4.0904	0.00026012
Age	0.0006	0.0006	0.1277	0.1678	0.9776	0.33537494
Male ratio	0.0079	0.0109	0.1108	0.1243	0.7198	0.47671474
HT	0.0036	0.0105	0.0506	0.0590	0.3396	0.73632372
CVD	0.0447	0.0125	0.4409	0.5290	3.5812	0.00108518
DM	0.0327	0.0186	0.2444	0.2922	1.7553	0.08848030
CNS	0.0149	0.0198	0.1120	0.1302	0.7541	0.45611313

*Temp* temperature, *HT* hypertension, *CVD* cardiovascular disease, *DM* diabetes mellitus, *CNS* central nervous system disorders, *RC* Regression coefficient, *SD* Standard deviation, *PCC* Partial correlation coefficient, *t* t-value, *p p*-value.

**Table 4:** The environmental temperature at which bath-related death are more likely to occur in each Kagoshima region with a focus on winter months.

City	n	Maximum temp		Minimum temp		Mean temp		Temp difference	
		°C	*p*	°C	*p*	°C	*p*	°C	*P*
A	300	14.5	0.0222	5.0	0.0143	9.0	0.0084	8.0	0.0173
B	79	12.5	0.0034	6.0	0.0254	9.0	0.0036	10.0	0.0790
C	32	14.5	0.0061	9.0	0.0215	9.0	0.0333	8.0	0.2572
D	47	12.0	0.0068	3.0	0.0064	7.5	0.0235	8.5	0.1213
E	65	13.5	< 0.0001	2.5	0.0279	7.0	0.0020	7.5	0.1679
F	75	12.5	0.0091	3.0	0.0076	9.5	0.0202	6.0	0.2211
G	22	13.5	0.0127	2.5	0.0053	9.0	0.0087	11.5	0.0179
H	71	13.5	0.0089	3.0	0.0271	7.5	0.0079	7.0	0.0351
I	61	13.0	0.0022	0.5	0.0069	5.0	0.0088	10.5	0.0333
J	127	13.0	0.0044	0.0	< 0.0001	5.0	< 0.0001	10.5	0.0042
K	25	9.0	0.0338	1.0	0.0295	4.5	0.0119	8.0	0.3123
L	49	14.0	0.0079	1.5	< 0.0001	5.0	< 0.0001	9.5	0.0174
M	32	11.0	0.0166	1.5	0.0099	6.0	0.0435	11.0	0.0480
N	67	14.5	0.0083	1.0	0.0499	7.0	0.0278	9.0	0.0307
O	18	15.5	0.0090	9.5	0.0094	11.5	0.0301	5.0	0.0270
P	9	14.5	0.0064	10.5	0.0077	9.5	0.0092	6.5	0.0669
Q	31	17.5	0.0071	11.5	0.0129	15.0	0.0591	5.5	0.3740
R	16	19.0	0.0460	13.0	0.0267	15.5	0.0333	5.0	0.6039
S	7	17.0	0.0357	12.5	0.0299	15.5	0.0355	4.5	0.4120

Values are presented as the cut off of mortality rate of each degree in Kagoshima region (A, Kagoshima city; B, Ibusuki and Minamikyushu cities; C, Makurazaki city; D, Minamisatsuma city; E, Hioki and Ichikikushikino cities; F, Satsumasendai city; G, Satsuma town; H, Akune and Izumi cities; I, Isa city and Yokogawa town; J, Aira and Kirishima cities; K, Soo city; L, Shibushi city; M, Kimotsuki and Kinko towns; N, Kanoya city; O, Tanegasima (island); P, Yakushima (island); Q, Amamioshima (island); R, Tokunoshima (island); S, Okinoerabu (island)) and are shown as maximum, minimum, mean temperature and temperature difference within a day (maximum temperature—minimum temperature). Temp, temperature; *p*, *p*-value.

## Discussion

During 2006–2019, there were 29,406 inquest cases in Kagoshima Prefecture. Among them, 2689 (9.1%) were cases of bath-related death. The number of bath-related deaths was approximately 2.8 times the number of automobile accidental deaths, and, therefore, bath-related death in Kagoshima Prefecture must comprise a relatively large percentage of all deaths. Moreover, the crude mortality rate (per 100,000 population) of bath-related death in Kagoshima Prefecture is estimated to be 8.9–13.4 (mean 11.4). This rate is similar to that in other prefectures of Japan including one in the north (8.3–10.0)^1–7^. On the other hand, the crude mortality rate of bath-related death in the United States (U.S.) is reported to be 0.16^15^, and the rate in Kagoshima Prefecture is approximately 61-fold higher. In addition, the age groups in which bath-related death occur are clearly different between the U.S. and Kagoshima Prefecture. Bath-related death is most common among children < 5 years old in the U.S.^15^, with most deaths occurring as accidental drowning while playing in the bathtub. However, our results show that bath-related death is most common among those aged ≥ 65 years (90% of all deaths) in Kagoshima Prefecture, with most deaths attributed to sudden natural causes such as ischemic heart disease. Accordingly, since the causes of bath-related death are fundamentally different between the U.S. and Japan, prevention strategies should be developed that are specific to Japan to reduce the number of bath-related deaths in Japan.

Our results indicate that bath-related death occurs most frequently in those aged > 65 years and during the cold winter season, which is consistent with results reported previously from other prefectures in Japan^1–7^. Many bathrooms and other rooms in Japan are unheated, even in winter, and the cold temperatures have been considered to contribute to bath-related death^8^. The Japanese bathing style is to immerse the body in hot water (> 41 °C) and elderly persons in Japan take a bath almost every day, especially in the winter^9^. Studies have indicated that patients diagnosed with angina pectoris may show electrocardiogram (ECG) changes that indicate ischemic changes or arrhythmias, such as ventricular tachycardia, supraventricular extrasystole, ventricular extrasystole, and/or a tendency of bradycardia, during usual bathing^7,10^. These arrhythmias, in particular ventricular tachycardia, may trigger a fatal event while in the bathtub. Thus, elderly people in Japan may have some undiagnosed underlying diseases that are risk factors of bath-related death.

In the present study, hypertension, a significant risk factor for cardiovascular mortality, was the most common past health issue among bath-related death cases. Several physiological studies have demonstrated that elderly people with hypertension often show a sudden decrease of blood pressure, followed by cardiopulmonary arrest^1,5^. The results of those studies, as well as our results suggest that cardiac events may occur more frequently in people with hypertension than in healthy individuals during bathing.

Our findings also indicate that most bath-related death occurs from 16:00 to 20:00, a time the elderly usually bathe in Japan, with most deaths occurring in the home bathroom. While alcohol consumption prior to bathing is a risk factor for bath-related death, only 4.3% (115 of 2689 cases) of our cases of bath-related death had consumed alcohol prior to taking a bath^4,5,16,17^. Therefore, bath-related death occurs frequently in the elderly without alcohol consumption.

Our results showed that the incidence of bath-related deaths was overwhelmingly higher among those living alone than among those living with their families. People who live alone take baths in a cold bathroom where nobody has bathed before them. In addition, there is no one who can help them when they have serious accidents while bathing. From a preventive perspective, elderly people, especially those with risk factors such as cardiac disease or hypertension should live with their family if possible. Even if they cannot live together, the elderly should be supervised by their neighbors and health visitors. In addition, the installation of remote monitoring systems should be considered, if possible. Recently, there have been engineering attempts in the bathroom to prevent bath-related deaths. For instance, a system has been devised to prevent drowning in the bathtub by installing a device on the bathroom wall that monitors ECG and respiratory status^18^, and interlocking it with a relief device (such as automatically draining the water in the bathtub) when aforementioned dangerous change in ECG or respiratory status that could lead to a bathing accident is detected.

As expected, very few of the bathing deaths were subjected to forensic autopsy (29 cases, 1.08%). In the majority of cases where no autopsy was performed, the cause of death was diagnosed presumptively with reference to the external findings, postmortem CT findings, and past history of illness. Drowning is diagnosed if there is evidence of froth, which is observed typically as a fungiform structure around the mouth and nostrils of the corpse^19,20^. Postmortem CT findings suggestive of drowning include fluid accumulation in the paranasal sinuses and the lower airways, diffuse pulmonary ground-glass opacities, pleural effusion, and gastric distention and contents, none of which are specific to drowning^21,22^. As the ischemic myocardium cannot be detected by postmortem CT, the diagnosis of suspected ischemic heart disease is often made by detecting severe calcification of the bilateral coronary arteries or stenosis/occlusion of the coronary arteries by CT angiography (although the latter is not generally performed)^23^. It must be stressed that it is difficult to diagnose the correct cause of death in case of bath-related death, including, but not limited to the cause of drowning, from external examination and postmortem CT alone. Therefore, it cannot be considered an appropriate statistic for the cause of death in bath-related deaths. Although the number is small, most of the deaths in the autopsy cases were due to drowning. In 9 of the 24 cases diagnosed as drowning, the cause of death could be determined by autopsy, and the most common cause was cardiovascular disease. According to a study of autopsy cases in the 23 wards of Tokyo where there is a medical examiner system, more than half (54.5%) of the bath-related deaths had pathological findings indicating cardiovascular disease, but 18.4% had no findings (pathological findings, medicinal or toxic substances) that could lead to the cause of death^12^. Bath-related deaths are thought to be caused by a number of factors, and forensic autopsy is essential to determine the cause of death. Therefore, it is necessary to change the Japanese death investigation system from the current police-oriented system to a forensic pathologist-oriented system that truly emphasizes the investigation of the cause of death.

Our study demonstrates that environmental temperatures such as the daily maximum, minimum, and mean temperatures all had a much greater effect on the incidence of bath-related deaths than the other confounding factors including age, male ratio, prevalence of hypertension, cardiovascular disease, diabetes mellitus, and central nervous system disorders. We have been able to identify the environmental temperatures at which bath-related deaths are most likely to occur with focus on the winter season as follows: daily maximum temperature of 13.5 °C or lower, daily minimum temperature of 3.5 °C or lower, and daily mean temperature of 9.0 °C or lower. We believe that bath-related deaths can be prevented by issuing warnings (so-called "Bath-Related Death Alerts") to refrain from bathing on days when all environmental temperature conditions are met, especially during the cold winter months. The means of issuing warnings include our website, television, radio, and internet news. Since the daily maximum and minimum temperatures are known by 16:00 to 20:00, when Japanese people usually bathe, effective alerts could be issued. The best prevention strategy against bath-related deaths is to avoid bathing itself on dangerous days. Since Kagoshima Prefecture is long from north to south and the environmental temperature differs from region to region, warnings are meaningless unless they are issued on a regional basis. Actually, the temperature at which bath-related death was more likely to occur differed by region (maximum, 9.0–19.0 (median 13.5) °C; minimum, 0.0–13.0 (median 3.0) °C; mean, 4.5–15.5 (median 9.0) °C). The difference in each environmental temperature between regions may be due not only to the regional differences in confounding factors examined in this study (rate of aging, rate of males, prevalence of hypertension, cardiovascular disease, diabetes mellitus, and central nervous system disorders), but also to the differences in factors such as bathing habits (frequency, preferred water temperature, style of immersion), bathroom facilities, and so on, which were not examined here. In the future, we hope to establish a formula for calculating the incidence of bath-related deaths by conducting a multiple regression analysis that includes all of the confounding factors related to bathing, and to issue an alert that can calculate the risk of bath-related deaths at the individual level; “Tailor-made Bath-Related Death Alerts”. Therefore, if it can be confirmed that the number of bath-related deaths can be reduced through the use of alerts for bath-related deaths in Kagoshima Prefecture, it may be possible to use artificial intelligence to analyze big data on bath-related deaths nationwide.

This study has several limitations. First, the study retrospectively examined the occurrence of bath-related deaths in a limited area, Kagoshima Prefecture, and reported it as an epidemiological feature of bath-related deaths in general. This study showed that the epidemiological characteristics, including the environmental temperatures at which these deaths are most likely to occur, vary from region to region within Kagoshima Prefecture, and there must be further variations throughout Japan. This issue can be resolved in the future by analyzing data on bath-related deaths throughout Japan in cooperation with other institutions. However, this study is a challenging research to reach this goal. Second, the analysis of environmental temperatures at which bath-related deaths are likely to occur was calculated based on records from the past 14 years, and we cannot guarantee that the same will be true in the future. Therefore, it must be said that the time frame is also limited. We believe that continued prospective study will help us determine more accurately the environmental temperatures at which bath-related deaths are likely to occur. Since it was difficult to obtain information on the temperature at the time the bath-related death occurred, we estimated the temperature at which bath-related deaths were likely to occur based on the environmental temperature (maximum, minimum, mean temperature, etc.) on the day of occurrence. In a strict sense, this does not mean that the temperature at which bath-related deaths are likely to occur has been identified. However, we believe that the method used in this study was appropriate for our purpose because it is better to predict the temperature based on the maximum, minimum, and mean temperatures of the day, as described above, in order to issue an alert to prevent bath-related deaths.

In conclusion, it is highly likely that bath-related deaths are closely related to the Japanese unique bathing culture. In addition, multiple factors are involved in bath-related deaths, and forensic autopsies are essential in order to elucidate the pathogenesis and mechanism of the death. However, the current Japanese death investigation system makes it difficult to increase the number of autopsies on bath-related deaths rapidly, so the first priority is to reduce the number of bath-related deaths in Kagoshima Prefecture by issuing alerts based on the results of this study, especially during cold winter months. In order to establish the alert, this study focused on the environmental temperature, which has the greatest influence on the occurrence of bath-related deaths. We believe that we have obtained basic data for the establishment of alerts. If the alert is successful, Kagoshima Prefecture could be used as a model case for nationwide implementation.

## Material and methods

All inquest records (n = 29,406) were collected for deaths during the period of 2006–2019 in Kagoshima Prefecture with the cooperation of the First Department of Criminal Investigation of the Kagoshima Prefectural Police Headquarters. We retrospectively reviewed the inquest records of cases that died unexpectedly in the bathroom while bathing, and extracted inquest records involving death that occurred in the bathroom, including death in spas and other bathing facilities. The records of sudden death in the bathroom were carefully gathered and assessed. In each case, we reviewed parameters, such as age, sex, date of incidents, place of death (home, spa, other bathing facilities), place of incidents in the bathroom (bathtub, space next to a bathtub, dressing room, sauna), living style (alone, with family), a history of alcohol use before bathing, a past history of illness, cause of death, and the area where individual cases lived (subdivided into 19 regions based on the jurisdiction of the police station in Kagoshima Prefecture). However, the cause of death is only an estimate as forensic autopsy were not carried out in the majority of cases. In order to examine the relationship between the occurrence of bath-related deaths and environmental temperature, we extracted the environmental temperature (maximum, minimum, mean, temperature difference within a day) on the date of death from the Kagoshima Meteorological Observatory website (https://www.jma-net.go.jp/kagoshima/).

All figures and plots were generated using Adobe Photoshop Elements 2020 software based on data calculated in Excel Office 2019.

Statistical analysis was performed using chi-square (χ^2^) test under a binomial distribution of counts for comparisons between men and woman in total numbers and age-specific mortality rate. The Mann–Whitney U test was used to compare the time required from death to discovery for men and women. The correlation coefficient of each temperature and mortality rate was determined using Spearman’s correlation coefficient by rank test. Multiple regression analysis of confounding factors in the relationship between the incidence of bath-related deaths and each environmental temperature was carried out. The Receiver Operating Curve (ROC curves) for the incidence of bath-related deaths at each environmental temperature was developed, and cut-off values for the temperature at which bath-related deaths are likely to occur were calculated. A value of *p* < 0.05 was considered to indicate a significant difference. All statistical analyses were performed using Bell Curve for Excel software under the supervision of a medical statistician.

This study was approved by the Ethics Committee for Epidemiological Research, Graduate School of Medical and Dental Sciences, Kagoshima University (Approval No. 200248) and was carried out in accordance with the Declaration of Helsinki Principles. Moreover, this study was conducted using inquest records from the past, and we could not obtain informed consent from the bereaved family for the use of the records. Therefore, in accordance with the "Ethical Guidelines for Medical Research Involving Human Subjects (enacted by the Ministry of Health, Labor and Welfare in Japan)," Section 12-1 (2) (a) (c), information on the implementation of the study was posted on our website (http://www.kufm.kagoshima-u.ac.jp/~legalmed/), and if there was a request to refuse the use of the samples for research, they were excluded from samples of this study. In addition, the Ethics Committee for Epidemiological Research, Graduate School of Medical and Dental Sciences, Kagoshima University (Approval No. 200248) has approved the waiver for the informed consent of this study.

## Supplementary Information

Supplementary Table 1.

## Data Availability

The data that support the findings of this study are available from the corresponding author upon reasonable request.
