# Supplementary material for: Development of prevention strategies against bath-related deaths based on epidemiological surveys of inquest records in Kagoshima Prefecture
Source: Sci Rep. 2023 Feb 8;13:2277. doi: 10.1038/s41598-023-29400-7 (PMC9908979; doi:10.1038/s41598-023-29400-7)
Supplement: Supplementary file 1 — Supplementary Table 1. [file 41598_2023_29400_MOESM1_ESM.docx]

Supplemental Table 1. The occurrence frequency of bath-related death in each Kagoshima region.

|  |  | Maximum temp | | Minimum temp | | Mean temp | | Temp difference | |
| --- | --- | --- | --- | --- | --- | --- | --- | --- | --- |
| City | n | C.C. | *p* | C.C. | *p* | C.C. | *p* | C.C. | *p* |
| A | 580 | -0.9157 | <0.0001 | -0.9550 | <0.0001 | -0.9241 | <0.0001 | 0.8122 | <0.0001 |
| B | 151 | -0.8595 | <0.0001 | -0.6675 | <0.0001 | -0.7696 | <0.0001 | -0.1557 | 0.43 |
| C | 44 | -0.8667 | <0.0001 | -0.6945 | 0.00087 | -0.5667 | 0.0046 | 0.4193 | 0.075 |
| D | 71 | -0.8326 | <0.0001 | -0.5922 | 0.00081 | -0.8151 | <0.0001 | 0.3122 | 0.13 |
| E | 129 | -0.7709 | <0.0001 | -0.7663 | <0.0001 | -0.8406 | <0.0001 | 0.2823 | 0.12 |
| F | 140 | -0.8164 | <0.0001 | -0.7232 | <0.0001 | -0.6623 | <0.0001 | 0.2975 | 0.098 |
| G | 51 | -0.6369 | 0.00016 | -0.2823 | 0.105 | -0.5807 | 0.00102 | 0.1609 | 0.44 |
| H | 134 | -0.7439 | <0.0001 | -0.8651 | <0.0001 | -0.8131 | <0.0001 | 0.6174 | 0.0025 |
| I | 117 | -0.8235 | <0.0001 | -0.7132 | <0.0001 | -0.6901 | <0.0001 | 0.4191 | 0.016 |
| J | 245 | -0.8634 | <0.0001 | -0.8910 | <0.0001 | -0.8443 | <0.0001 | 0.5893 | 0.00049 |
| K | 54 | -0.6323 | 0.00043 | -0.6323 | 0.00043 | -0.7130 | <0.0001 | 0.0126 | 0.95 |
| L | 84 | -0.7651 | <0.0001 | -0.7546 | <0.0001 | -0.8171 | <0.0001 | 0.5077 | 0.011 |
| M | 63 | -0.8666 | <0.0001 | -0.6315 | 0.0025 | -0.6644 | 0.0038 | 0.2842 | 0.23 |
| N | 139 | -0.6373 | <0.0001 | -0.6644 | <0.0001 | -0.6957 | <0.0001 | 0.3460 | 0.054 |
| O | 36 | -0.8451 | <0.0001 | -0.6200 | 0.0019 | -0.7391 | 0.00053 | 0.2485 | 0.46 |
| P | 15 | -0.8182 | 0.0046 | -0.4209 | 0.14 | -0.8462 | 0.005 | 0.1429 | 0.71 |
| Q | 55 | -0.8659 | <0.0001 | -0.7458 | 0.0006 | -0.8052 | <0.0001 | 0.5737 | 0.0149 |
| R | 35 | -0.6354 | 0.0019 | -0.7487 | 0.00045 | -0.4545 | 0.042 | 0.5110 | 0.077 |
| S | 15 | -0.9066 | 0.0017 | -0.9441 | 0.0017 | -0.9429 | 0.00068 | -0.0714 | 0.86 |

Values are presented as the mean standard deviation of mortality rate of each degree in Kagoshima region (A~S; supported Fig. 6) and are shown as maximum temperature, minimum temperature, mean temperature and temperature difference within a day (maximum temperature - minimum temperature). Temp, temperature; C.C., correlation coefficient; *p*, *p*-value.
